# Quantification of the effects of antibodies on the extra- and intracellular dynamics of *Salmonella enterica*

**DOI:** 10.1098/rsif.2012.0866

**Published:** 2013-02-06

**Authors:** Olivier Restif, Yun S. Goh, Matthieu Palayret, Andrew J. Grant, Trevelyan J. McKinley, Michael R. Clark, Pietro Mastroeni

**Affiliations:** 1 Department of Veterinary Medicine, University of Cambridge, Madingley Road, Cambridge CB3 0ES, UK; 2 Novartis Vaccine Institute for Global Health, Via Florentina 1, 53100 Siena, Italy; 3 Department of Chemistry, University of Cambridge, Lensfield Road, Cambridge CB2 1EW, UK; 4 Department of Pathology, University of Cambridge, Tennis Court Road, Cambridge CB2 1QP, UK

**Keywords:** bacteriology, *Salmonella enterica*, infection dynamics, antibodies, mathematical model, likelihood

## Abstract

Antibodies are known to be essential in controlling *Salmonella* infection, but their exact role remains elusive. We recently developed an *in vitro* model to investigate the relative efficiency of four different human immunoglobulin G (IgG) subclasses in modulating the interaction of the bacteria with human phagocytes. Our results indicated that different IgG subclasses affect the efficacy of *Salmonella* uptake by human phagocytes. In this study, we aim to quantify the effects of IgG on intracellular dynamics of infection by combining distributions of bacterial numbers per phagocyte observed by fluorescence microscopy with a mathematical model that simulates the *in vitro* dynamics. We then use maximum likelihood to estimate the model parameters and compare them across IgG subclasses. The analysis reveals heterogeneity in the division rates of the bacteria, strongly suggesting that a subpopulation of intracellular *Salmonella*, while visible under the microscope, is not dividing. Clear differences in the observed distributions among the four IgG subclasses are best explained by variations in phagocytosis and intracellular dynamics. We propose and compare potential factors affecting the replication and death of bacteria within phagocytes, and we discuss these results in the light of recent findings on dormancy of *Salmonella*.

## 1. Introduction

Over the last 15 years, the use of mathematical models to complement traditional statistical analyses of experimental data in microbiology has generated new insights on the population dynamics of pathogens inside their hosts [1–4]. An overarching goal of many of these studies is to estimate the relative roles of resource limitation and immune responses in controlling the growth and spread of an infection. While this question has traditionally been considered at the level of the whole host, modern observation techniques have started to unravel variations in pathogen growth within individual infected cells. In particular, there is mounting evidence that antibodies present in the serum can directly affect the intracellular dynamics of bacteria, such as *Listeria monocytogenes* [5], *Legionella pneumophila* [6] or *Salmonella enterica* [7]. All these studies measured net changes in pathogen numbers. In order to make inferences on the concurrent processes underlying these changes (e.g. replication, death or migration of pathogens), mathematical models need to be developed alongside experimental observations, and fitted to the data using appropriate statistical tools. This approach typically provides two quantitative outcomes: a ranking of alternative mechanistic scenarios (based on the relative goodness of fit of the corresponding alternative models), and numerical estimates of the parameters of the models. An important caveat is that predictions from such models cannot provide definitive proof for the existence of any unobserved mechanism, but they can guide further experimental investigation in a more focused and efficient way.

*Salmonella enterica* serovar Typhimurium (*S*. Typhimurium), although widely used as a laboratory model for the study of typhoid-like infection in mice, is also an important human pathogen. A major source of food poisoning around the world, *S.* Typhimurium also causes bacteraemia in immuno-compromised patients, such as malaria and AIDS patients and in African children under 2 years of age [8,9]. *Salmonella enterica* is a facultative intracellular pathogen; a key virulence determinant of the bacteria is the ability to grow and persist within phagocytes [10,11]. Despite its intracellular niche, *S. enterica* spreads rapidly from phagocyte to phagocyte within the liver and spleen during the acute phase of infection [12]. This finding was made possible by the development of novel methods, combining fluorescence microscopy which allows the counting of bacteria within individual macrophages, and mechanistic mathematical models which allow inferences to be drawn from unobserved processes. Further knowledge of the intimate interactions between *S.* Typhimurium and individual macrophages can be gained by fitting models to data obtained from tailor-made *in vitro* experiments. In a recent study, Gog *et al.* [13] combined several observation and inference techniques to quantify various factors affecting phagocytosis rates within murine macrophage cultures.

Antibodies have long been known to play an important role in mediating protective immunity against infection by *S. enterica* [14,15], but the actual mechanisms at the cellular level are only beginning to emerge. Opsonization (the process of antibodies present in serum binding to antigens) of *S. enterica* with immune serum has been shown to increase not only uptake by macrophages, but also intracellular bactericidal activity, both with serovar Typhi using human serum [16] and with serovar Typhimurium using murine serum [7]. Although the concentrations of immunoglobulins (Ig) G and M in human serum have been shown to correlate positively with oxidative burst against invasive strains of *S.* Typhimurium [17], the specific roles of the different immunoglobulins involved remain unclear [16]. We set out to investigate the role of IgG in mediating the interaction between *S*. Typhimurium and human macrophages, with the ultimate end to help the development of new treatments against non-typhoid salmonellosis. Using *in vitro* cultures of human macrophages, we recently demonstrated that different IgG subclasses affect the phagocytosis rate of *S.* Typhimurium differently, through Fcγ receptors [18]. We decided to extend that study by analysing the effect of prior opsonization with different IgG subclasses on the intracellular dynamics of *S*. Typhimurium following phagocytosis into human cells *in vitro*.

Here we associate a mathematical model with new experimental data on intracellular bacterial counts in order to determine the factors that modulate bacterial replication and mortality inside macrophages within 9 h of infection with *S*. Typhimurium opsonized with human IgG isotypes 1, 2, 3 or 4. These four subclasses, numbered according to their relative abundance in human serum, are known to differ in their affinity to Fc receptors on phagocytic cells, and are therefore expected to produce different dynamics following opsonization. In addition, the large amount of information present in the experimental data (distributions of bacteria in samples of 450 infected cells at two time points and in five opsonization groups) allows us to explore and assess the value of several hypotheses concerning the effects of antibodies on the intracellular replication and death of *Salmonella* bacteria, which had been suggested by previous empirical and theoretical studies. Our results reveal substantial heterogeneity among the intracellular bacteria and far-reaching effects of different antibody subclasses.

## 2. Material and methods

### 2.1. Bacterial strains, antibodies and cell culture

The bacterial strain used in the study is a green fluorescence protein (GFP)-expressing *S.* Typhimurium SL3261 with a short peptide-coding sequence inserted into its *ompA* gene [18]. The short peptide, with sequence TSSPSAD, is a mimotope of the human CD52 antigen. Expression of the peptide in the OmpA protein allows tagging of the OmpA protein with a panel of humanized CD52 antibodies. The humanized anti-CD52 antibodies share the same variable regions (CAMPATH-1 [19]) that recognize the human CD52 mimotope, but are of different human antibody subclasses, either IgG1, IgG2, IgG3 or IgG4 [20,21]. The non-specific control antibody used is the recombinant human Fog-1 IgG1 antibody [21] which recognizes the human RhD antigen. The phagocytes used in this study belong to the human monocyte cell line THP-1. The cells were grown in RPMI-1640 supplemented with 10 per cent foetal calf serum, 2 mM l-glutamine, 0.05 mM 2-mercaptoethanol at 37°C. Prior to bacterial infection, THP-1 cells were grown in RPMI-1640 supplemented with 10 per cent Nu serum (VWR), 2 mM l-glutamine, 0.05 mM 2-mercaptoethanol for 22 days, followed by an incubation with 100 U ml^–1^ rIFNγ for 48 h [18,22].

### 2.2. Bacterial opsonization and infection

These were performed as previously described by Goh *et al.* [18]. Briefly, opsonization of overnight bacterial culture was performed by incubation in either the humanized anti-TSSPSAD antibodies (IgG1, IgG2, IgG3 or IgG4) or the non-specific control antibody at 37°C with shaking for 30 min. The dilution of the antibodies for opsonizing bacteria was determined as the lowest dilution that does not cause bacterial agglutination, which corresponded to 25 µg ml^−1^. THP-1 cells were then exposed to the opsonized bacteria at multiplicity of infection of 10 bacteria per THP-1 cell for 45 min. Hereafter, the end of this period of exposure is taken as the initial time point (*t* = 0). The infected cells were incubated with fresh culture medium containing 100 μg ml^−1^ gentamicin for an hour to kill any remaining extracellular bacteria, at which point half of the cell cultures were harvested for further analysis (first data time point, *t* = 1 h). In the remaining cultures, the medium was replaced with fresh medium supplemented with 10 μg ml^−1^ gentamicin and the cells were incubated for another 8 h (second data time point, *t* = 9 h).

### 2.3. Visualization of intracellular bacteria

As previously described [18], THP-1 cells were plated onto poly-l-lysine-treated coverslips (Fisher Scientific) 12 h prior to infection. At *t* = 1 h and *t* = 9 h, THP-1 cells were fixed with 4 per cent paraformaldehyde for 15 min, and then incubated with mouse monoclonal O4 antibody (Abcam) and secondary goat anti-mouse Alexa Fluor 405 antibody (Invitrogen). Each antibody reagent was diluted 1 : 1000 in 10 per cent normal goat serum (Dako). After the coverslips were mounted onto Vecta bond-treated glass slides (Vector Laboratories) with Vectashield mounting medium (Vector Laboratories), they were examined using fluorescence microscopy (Leica DM6000B). Intracellular bacteria were discriminated from extracellular bacteria by the presence of GFP and the absence of labelling by the mouse monoclonal O4 antibodies. The experiment was performed three times with each of the four specific antibody types and the one control antibody. In each replicate, 450 infected cells were examined in order to determine the distribution of intracellular bacteria. Only a few extracellular bacteria were observed across all samples, indicating that re-infection events should be extremely rare. The complete dataset is presented in the electronic supplementary material, table S1.

### 2.4. Mechanistic model

Following on from previous work on cell-level dynamics of *S*. Typhimurium infection [12,13], we described the dynamics of the system by a set of differential equations representing temporal variations in the numbers of cells harbouring different numbers of bacteria. In line with the experimental protocol, we assumed that a large number of uninfected cells were exposed to opsonized bacteria for 45 min, when phagocytosis could occur, and from then onwards all extracellular bacteria were killed. Thus, we started with a baseline model containing four parameters. The effective division rate of a replicating bacterium in a cell containing *i* bacteria is modelled as *a* exp(–*b i*) where *a* is the maximum division rate and *b* measures density-dependent reduction in division rate (accounting for limitation of accessible resources in the *Salmonella*-containing vacuole [12]). Bacteria are degraded (and are no longer visible) at rate *d*; as explained in the next paragraph, we also consider as a different process the possibility that bacteria stop replicating (and possibly die) while remaining visible. The phagocytosis rate *Φ*(*t*) is set equal to a constant *φ* for the first 45 min (−0.75 h < *t* < 0) and to zero afterwards (*t* > 0). Note that the assumption of a constant phagocytosis rate is justified by the large excess of bacteria in the medium (10 times the number of cells) and the short duration of exposure.

**Figure 1.**
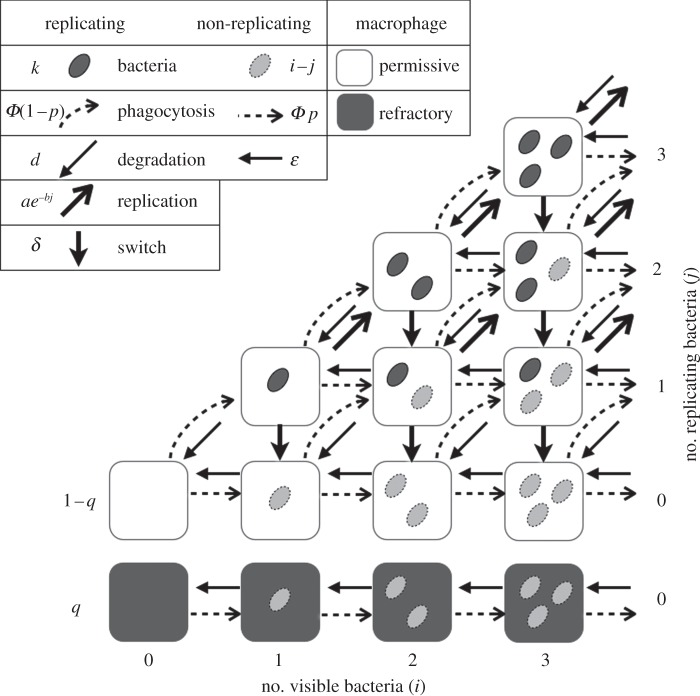
Schematic of the complete mechanistic model showing the different states of macrophages (boxes) based on the number of intracellular replicating and non-replicating bacteria, and the six transitions (arrows) between these states. The symbols next to the arrows in the legend represent the rates of the corresponding transitions per bacterium. The ‘switch’ from replicating to non-replicating, at rate *δ*, corresponds to scenario (i); phagocytosis of non-replicating bacteria, in proportion *p*, to scenario (ii); and refractory macrophages, in proportion *q*, to scenario (iii). For simplicity, cells containing more than three bacteria are not shown. Phagocytosis (dashed arrows) occurs during the first 45 min only.

**Figure 2.**
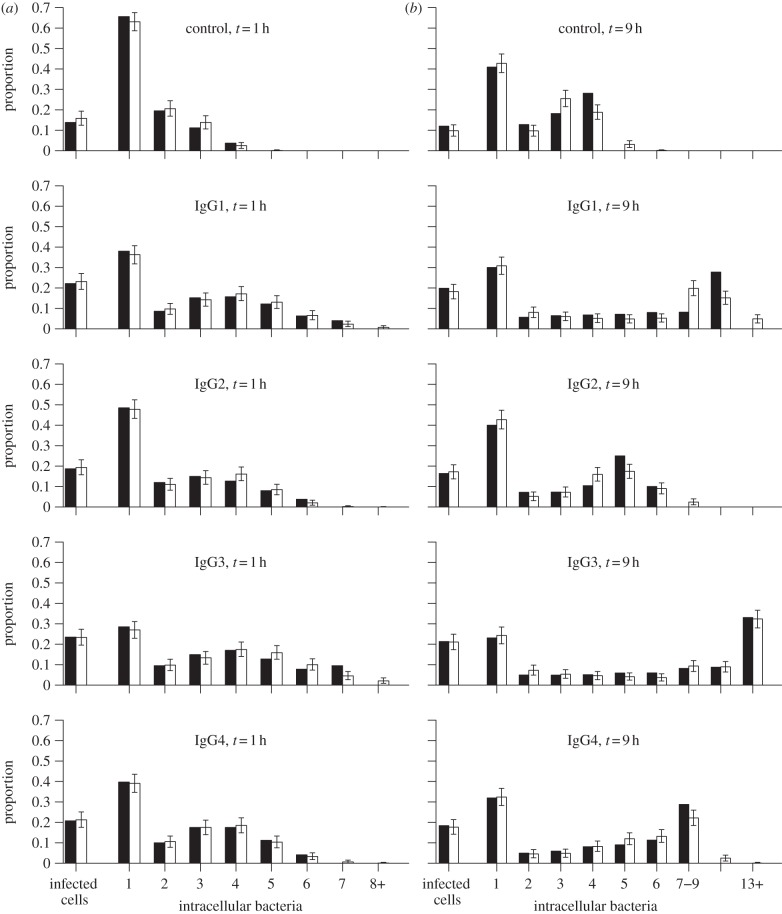
Proportion of infected cells and distribution of intracellular bacteria, at 1 h (*a*) or 9 h (*b*) after inoculation, for each antibody treatment (rows): experimental data (solid bars), predictions from fitted model (white bars) using the model with lowest AIC for each group (as per table 2). Error bars show the 95 per cent inter-quantile ranges from 10 000 Monte Carlo samples from the predicted distributions (see §2).

Preliminary data [18] indicated that the distributions of intracellular bacteria were bimodal: at the first time point with any of the four specific antibodies, a large proportion of infected cells contained a single bacterium, while there were more cells with three or four bacteria than cells with two bacteria. We show in §3 that this pattern is even more conspicuous at the second time point (figure 2). In line with a recent report [23], we hypothesized that the presence of a subset of non-replicating bacteria persisting within some macrophages might contribute to the observed distributions. To date, the potential mechanisms responsible for heterogeneity in bacterial replication are not known. Therefore, we sought to use our combined data and modelling framework to assess the credibility of three scenarios: (i) intracellular bacteria randomly enter a non-replicating state at rate *δ* and remain in that state until they are degraded; (ii) before phagocytosis, a proportion *p* of bacteria are in a non-replicating state (possibly induced by opsonization) and can be taken up by macrophages without distinction from active bacteria; (iii) a proportion *q* of macrophages (thereafter ‘refractory’ macrophages) are preventing intracellular bacteria from dividing. Unlike scenario (iii), the first two assume that infected cells can contain a mix of replicating and non-replicating bacteria (figure 1). In any case, non-replicating bacteria are assumed to remain visible by fluorescence microscopy (with no presumption as to whether they are effectively dead or in a dormant state) until they are degraded at rate *ɛ*. By including or excluding each scenario, we generated eight alternative models (table 2). In the following, ‘complete model’ refers to the inclusion of all three scenarios.

To account for these scenarios, we must assume that the observed intracellular bacteria fall into two classes: replicating and non-replicating. Specifically, the models keep track of a large number of variables *N_i_*_,*j*_(*t*) representing the number of ‘permissive’ macrophages that contain *i* visible intracellular bacteria, of which *j* are in a replicating state and *i–j* are non-replicating (according to scenarios (i) and (ii) above); and a second set of variables *M_i_*(*t*) that represent ‘refractory’ macrophages with *i* non-replicating bacteria, according to scenario (iii). Only with IgG3 did any cells contain more than 12 bacteria, most of which had up to 15. Hence we restricted the number of intracellular bacteria to 20 in our model for computational efficiency, but we checked with a few examples that allowing higher numbers did not affect noticeably the numerical output of the model. A schematic of the complete model, combining the three scenarios for non-replicating bacteria, is shown in figure 1. The dynamics of the permissive cells are described by the following set of differential equations (where dependence on time *t* has been omitted for brevity):
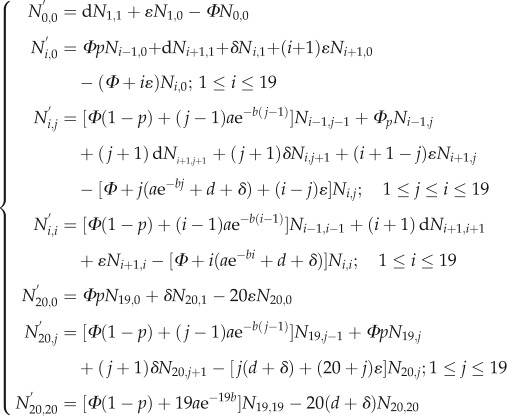


The dynamics of the refractory cells *M_i_* are described the following equation:
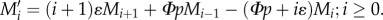


The numerical values of the eight parameters in this model (table 1) were unknown *a priori* and were estimated from the data using the statistical method described below. All equations in the system are linear, enabling us to solve them using matrix exponentials (see electronic supplementary material, methods).

**Table 1.** Definition of parameters used in the models.

symbol	definition
*a*	maximum replication rate of bacteria
*b*	coefficient of density-dependent reduction in bacterial replication
*d*	degradation rate of replicating bacteria
*δ*	rate at which intracellular bacteria switch to the non-replicating state
*ɛ*	degradation rate of non-replicating bacteria
*p*	initial proportion of non-replicating bacteria
*φ*	phagocytosis rate
*q*	proportion of refractory macrophages

### 2.5. Model fitting and parameter inference

In order to estimate the values of the parameters in our models, we computed the likelihood of the observed data (proportion of infected macrophages and distribution of intracellular bacteria numbers at 1 h and 9 h post-exposure, for each of the five opsonization treatments) given the theoretical distributions predicted by the model at the same two time points. We allowed the eight parameters to differ among the four IgG subclasses and the control, but we assumed they did not vary between experimental replicate, making a total of 40 parameters. Let *Θ_g_* = {*a_g_*, *b_g_*, *d_g_*, *δ_g_*, *ɛ_g_*, *p_g_*, *φ_g_*, *q_g_*} be the vector of parameters for antibody type *g* from 0 to 4 (where *g* = 0 represents the non-specific antibody and *g* = 1–4 represents each of the four IgG isotypes). Since the data comes from a full factorial design with two independently measured variables, two independent time points and five independent opsonization treatments, all in three independent replicates, the log-likelihood *LL*_*Θ*_ of the whole dataset is the sum of the five antibody-specific log-likelihoods *LL_g_* (0 ≤ *g* ≤ 4), each of which can be expressed as
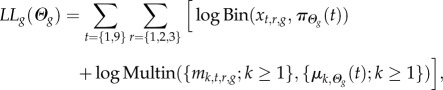
where subscript *r* stands for each of the three replicate experiments and *t* for time post-exposure (1 h or 9 h); *x_t,r,g_* is the observed number of infected cells, *π*_*Θ*_(*t*) the predicted proportion of infected cells according to the model, *m_i,t,r,g_* the observed number of macrophages with *i* bacteria and *μ_i_*_,*Θ*_(*t*) the predicted proportion of macrophages with *i* bacteria according to the model. Since the observed number of infected cells and the observed distributions of bacteria per macrophage were determined independently based on two samples of *S* = 450 macrophages within each replicate, we combined a binomial (Bin) and a multinomial (Multin) probability mass functions defined as follows:
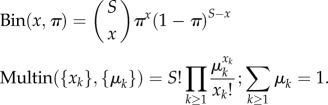
The predicted proportions (*π* and *μ_k_*) were derived from the numerical solution of the system of differential equations as follows:
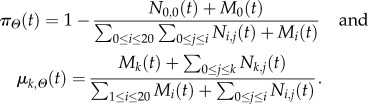


Each log-likelihood function *LL_g_* was maximized numerically using the built-in function *optim* in R [24], which implements the Nelder–Mead simplex algorithm; all maximizations were replicated with different initial conditions to reduce the risk of obtaining local maxima. This function also provides a numerical approximation of the Hessian matrix of the likelihood function around its maximum, which enabled us to calculate approximate 95% confidence intervals and correlation matrices for the parameters. Because parameters were constrained to be positive, correlation matrices could not be computed for parameters with a maximum-likelihood estimate (MLE) equal to zero (as the maximum is then on the edge of the domain over which the likelihood is defined); in that case, contour plots of the likelihood function provided a graphic assessment of the correlations.

In order to compare the relative importance of the three scenarios for non-replicating bacteria, we fitted eight different simplified versions of the model by setting the values of *δ*, *p* or *q* to zero. For example, scenario (i) alone corresponds to *p* = *q* = 0, leaving six parameters per group to estimate; while combining scenarios (ii) and (iii) corresponds to setting *δ* = 0 and estimating the remaining seven parameters per group. We compared these eight models within each opsonization group using Akaike's Information Criterion (AIC): 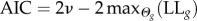 where *ν* is the number of parameters of the model considered. This allowed us to calculate weighted averages of the parameter estimates across the set of models, using the Akaike weight of each model *m* within the set *M* considered, defined by
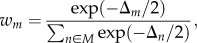
where *Δ_m_* is the difference between the AIC value for model *m* and the minimum AIC value within the set of models *M* [25]. In addition to these antibody-specific analyses, we fitted a second series of models to the whole dataset, whereby each parameter in turn was forced to have the same value either across all five antibodies or across the four specific IgG subclasses. We calculated the corresponding AIC values and weights using the total likelihood summed across all five opsonization groups.

Finally, we performed Monte Carlo sampling of the predicted distributions of cells to assess the goodness-of-fit of the models. Using the *rbinom* and *rmultinom* function in R, we generated 10 000 pseudo-random binomial samples of 450 cells (for the proportions of infected cells) and 10 000 multinomial samples of 450 infected cells (for the distributions of intracellular bacteria); the expected probabilities of the binomial and multinomial distributions were given by the numerical solutions of each fitted model at either *t* = 1 h or *t* = 9 h. We plotted the 2.5–97.5% inter-quantile range of the Monte Carlo simulated distributions alongside the observed data to highlight any deviation from the predicted values unlikely to be due to sampling noise.

## 3. Results

### 3.1. Observed distributions of intracellular bacteria

As previously reported [18], fluorescence microscopy observations at the first time point (1 h post-exposure) revealed differences in the proportions of infected macrophages and in the distributions of intracellular bacteria between opsonizing antibody subclasses (figure 2*a*). The cell cultures that were left to incubate for 9 h post-exposure showed slightly but consistently lower proportions of infected macrophages and larger numbers of intracellular bacteria (figure 2*b*). Both measurements at the two time points followed the same hierarchy among the different opsonization groups: bacteria opsonized with the non-specific IgG (control group) infected fewer macrophages and reached lower intracellular numbers than those opsonized with specific IgG2, followed in increasing order by IgG4, IgG1 and IgG3.

The distribution of intracellular bacteria at the first time point exhibits a weak bimodal pattern among the four specific IgG subclasses (revealed by the low proportion of infected cells containing two bacteria compared with lower and higher numbers in figure 2). The bimodal pattern is much more pronounced at the later time point across all five groups: while at least 35 per cent (and up to 62%) of infected cells contained five or more bacteria, large proportions (between 23 and 41%) contained only one bacterium. We used this pattern as a hallmark to assess the quality of our mathematical models. Even though the higher mode apparent in the experimental data may have been inflated by the grouping of cells containing large numbers of bacteria (which was deemed necessary due to possible inaccuracies in bacterial counts in highly infected cells), we used the same grouping for the predicted distributions when fitting the model to the data, hence avoiding any bias in our analysis.

### 3.2. Comparison of mechanistic models

We explored a range of hypotheses regarding the mechanisms underlying the observed distributions of intracellular bacteria and their variations across antibody treatments, as detailed in §2.5. In the absence of previous information about expected differences between IgG subclasses (apart from rates of phagocytosis), we followed two steps. First, we looked for qualitative differences between the five experimental groups (control, IgG1, IgG2, IgG3 and IgG4) by comparing the possible scenarios for non-replicating bacteria. Then we assessed quantitative variations between antibodies by fitting a series of simplified models to the whole dataset, each assuming that some parameter was invariant across the groups.

The baseline model, which includes phagocytosis, intracellular bacterial replication and death and assumes that all bacteria can replicate, fails to reproduce the observed bimodal distributions (see the electronic supplementary material, figure S1). Statistically, this model receives no support at all for any IgG subclass, compared with the other models considered (table 2). In total, eight models, obtained by including or excluding each of the three scenarios for non-replicating bacteria, were fitted to the data for each antibody subclass separately. Including any combination of alternative scenarios resulted in a significant improvement based on likelihood values (see the electronic supplementary material, table S2). According to AIC, the observed distributions from the four IgG isotypes and the control are best described by different models (table 2): for the control and IgG2 (which have the lowest bacterial loads), the best model includes scenario (iii) only, assuming a certain proportion of ‘refractory’ macrophages inhibit intracellular replication of bacteria; whereas for IgG1, 3 and 4 the best model combines scenarios (i), whereby intracellular bacteria progressively stop replicating, and (ii), which assumes that a proportion of bacteria are incapable of replication from the onset. However, with IgG4, AIC values show good support for an alternative model combining scenarios (i) and (iii); in other words, the data obtained with IgG4 can be explained by assuming that either a proportion of non-replicating cells or a proportion of refractory phagocytic cells were present at the start of the experiment.

**Table 2.** Comparison of eight mechanistic models fitted to the distributions of intracellular bacteria for each subclass of IgG. Scenarios (i)–(iii) correspond to the three proposed mechanisms for non-replicating bacteria as explained in §2. Columns 4–8 show the *Δ*AIC values of the eight models fitted to each experimental group (control and IgG1 to 4).

scenarios	parameters set to 0	no. parameters	control	IgG1	IgG2	IgG3	IgG4
(i)–(iii)	none	8	4.0	2.0	4.0	2.0	1.5
(ii) and (iii)	*δ*	7	2.0	267.7	2.0	501.3	65.5
(i) and (iii)	*p*	7	2.0	16.3	2.0	4.8	1.0
(i) and (ii)	*q*	7	11.2	0.0	42.1	0.0	0.0
(iii)	*δ, p*	6	0.0	290.0	0.0	543.7	63.8
(ii)	*δ, q*	6	9.0	267.3	39.9	501.5	85.5
(i)	*p, q*	6	352.2	168.2	662.0	24.1	331.3
none	*δ, ɛ, p, q*	4	480.6	994.0	1118.8	968.4	1035.9

We then fitted further models to the whole dataset, assuming that some parameters were invariant either across all five antibodies or across the four specific subclasses. Based on AIC, the best model has a common value for the baseline replication rate *a* across all groups, closely followed by a model where the replication parameter *b* is invariant across the four specific antibodies (table 3) and then followed by the combination of these two models. Attempts to impose invariance on other parameters obtained little or no support from AIC; the only one within 10 units of the best model assumes invariance of the degradation rate of non-replicating bacteria (*ɛ*) among the four specific IgG subclasses.

**Table 3.** Comparison of simplified versions of the complete model fitted to the whole dataset, obtained by assuming that certain parameters are invariant either across all five antibody groups (including the control) or across the four specific IgG subclasses (excluding the control).

invariant across all groups	invariant across IgG1–4	parameters	*LL*	*Δ*AIC
*a*	none	36	−1116.40	0
none	*b*	37	−1115.61	0.42
*a*	*b*	33	−1119.81	0.82
none	*a*	37	−1116.26	1.71
none	*a, b*	34	−1119.76	2.71
none	none	40	−1115.23	5.65
none	*ɛ*	37	−1119.57	8.34
none	*φ*	37	−1124.51	18.22
*p*	none	36	−1128.30	23.80
none	*p*	37	−1129.33	27.86
none	*d*	37	−1130.50	30.20
*ɛ*	none	36	−1131.82	30.83
*φ*	none	36	−1135.80	38.79
none	*q*	37	−1136.63	42.45
*q*	none	36	−1141.29	49.77
*d*	none	36	−1157.66	82.51
*b*	none	36	−1162.40	91.98
none	*δ*	37	−1377.02	523.23
*δ*	none	36	−1506.41	780.02

Figure 2 shows the observed and predicted distributions of visible intracellular bacteria, using the best model for each experimental group (as per table 2); other supported models produced very similar distributions (see the electronic supplementary material, figure S2). The predicted values were obtained by adding replicating and non-replicating bacteria, following the assumption that they are undistinguishable by fluorescence microscopy. Despite some discrepancies in the proportions of heavily infected cells beyond the 95% Monte Carlo sampling intervals, the fitted model reproduces the bimodal distributions and the variations between the five experimental groups.

### 3.3. Parameter estimates

**Figure 3.**
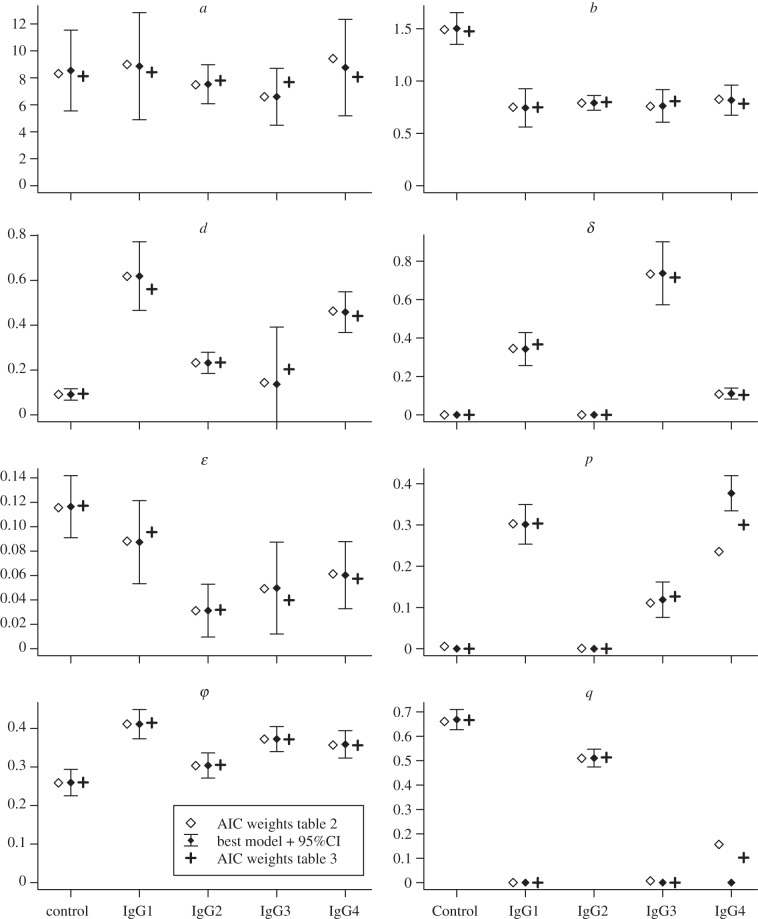
Maximum-likelihood estimates (MLE) for each parameter using different models. Open diamonds show AIC-weighted averages of MLE across the eight models shown in table 2 fitted to each opsonization group individually; filled diamonds with error bars show MLE and approximate 95% CIs from the best models within each opsonization group (as per table 2); crosses show AIC-weighted averages for all models listed in table 3, fitted to the whole dataset. Numerical values on vertical axes are expressed in h^−1^ for *a*, *d*, *δ*, *ɛ* and *φ*; *b* is dimension-less; *p* and *q* are proportions. See the electronic supplementary material, table S2 for a complete list of MLE by model.

The parameter estimates obtained by fitting each model to the data can be averaged using AIC weights. We produced two weighted averages, one for the set of models that were fitted to each opsonization group separately (table 2), and the other for the set of models fitted to the whole data. In the former case, the weight of every model is specific to each experimental group. As shown in figure 3, these two sets of estimates are generally very close. In addition, for each model, we generated approximate 95% confidence intervals on the parameter estimates, illustrated in figure 3 for the best model. These are deduced from the curvature of the likelihood function around its maximum for a given model, and reflect the information available in the data in support of each parameter. We can see in figure 3 that the variations across models are well within the level of uncertainty, with the notable exception of parameters *p* and *q* for IgG4 which will be discussed further down.

Given these uncertainties, the observed variations in the distributions of intracellular bacteria among antibody subclasses are best explained by a combination of qualitative differences in the replication regimes of *Salmonella* (governed by parameters *δ*, *p* and *q*) and quantitative variations in other processes—mainly phagocytosis (*φ*) and degradation (*d* and *ɛ*) of intracellular bacteria (figure 3). As expected from the observation of intracellular bacterial distributions at the first time point [18], the model predicts that all four specific antibodies enhance the rate of phagocytosis, at most by 58 per cent (IgG1 versus control). Note that the estimated rates of phagocytosis are slightly higher for IgG1 than IgG3 (0.41 and 0.37, respectively, albeit with overlapping confidence intervals), whereas our previous report [18] made the opposite prediction based on proportion of infected cells and bacterial counts. According to our model, this discrepancy is due to the substantial variations in bacterial killing rates (*d*): indeed, the half-life of intracellular replicating bacteria (given by the formula log 2/*d*) following opsonization with IgG1 is 80 per cent shorter than with IgG3.

As stated earlier, the only model compatible with the distributions observed following opsonization with either IgG1 or IgG3 combines scenarios (i) and (ii). Nine hours post-infection, these two groups exhibited the greatest numbers of bacteria per cell. Quantitatively, opsonization with IgG3, which had the highest bacterial loads, can be explained by a low degradation rate (*d*) of replicating bacteria, a relatively low initial proportion of non-replicating bacteria (*p* ≈ 10%, against 30% with IgG1), and a high rate of conversion from replicating to non-replicating (*δ*), twice as high as with IgG1. In contrast, opsonization with the non-specific antibody or with IgG2 is best described by scenario (iii) only. However, our parameter estimates for these two groups differ in many respects (figure 3). When considering the parameters common to all models (*a*, *b*, *d*, *ɛ* and *φ*), IgG2 is more similar to other IgG subclasses than to the control. Finally, IgG4 appears to be somewhere between the others, as we were not able to explain the observed data unambiguously with a single model. Although we can be confident that opsonization with IgG4 results in a low conversion rate (*δ*), the proportion *q* of refractory cells and the initial proportion of non-replicating bacteria *p* are more uncertain.

Univariate confidence intervals (figure 3), must be interpreted with caution when parameter estimates are correlated. We conducted pairwise analyses of parameters using covariance matrices (excluding parameters with an MLE equal to zero; see the electronic supplementary material, table S3) and contour plots of the likelihood function for the complete, eight parameter, model fitted to each opsonization group (see the electronic supplementary material, figure S3). The only systematically high correlation across all groups was between *a* and *b* (ranging between 0.78 and 0.95); this could be expected as these parameters affect the replication rate in opposite ways. Although correlations between *p* and *q* could not be computed, graphically the two parameters appeared to be negatively associated (see the electronic supplementary material, figure S3), meaning that it is possible to trade a small proportion of non-replicating bacteria for a small proportion of refractory cells (by varying the values of *p* and *q* in opposite directions around the MLE) without decreasing substantially the likelihood, especially with IgG4.

### 3.4. Predicted dynamics

**Figure 4.**
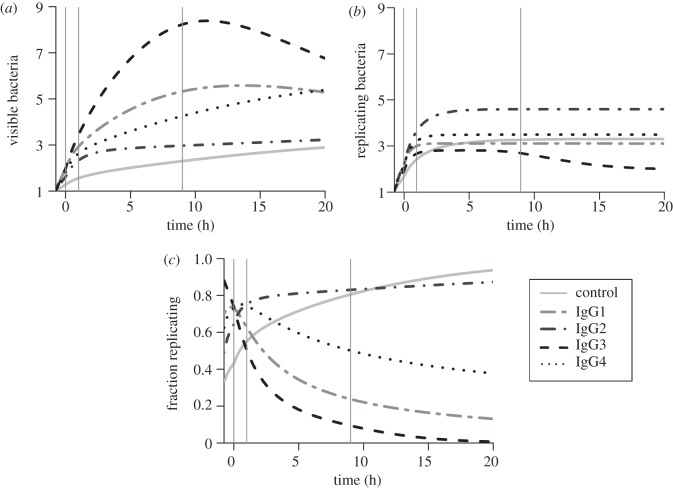
Predicted temporal variations in the mean numbers of bacteria per infected cell. (*a*) Mean numbers of visible bacteria among all infected cells. (*b*) Mean numbers of replicating bacteria in cells containing at least one replicating bacterium; this excludes refractory cells, hence the relatively high numbers in the control and IgG2. (*c*) Proportion of all visible bacteria that are in the replicating state, including all infected cells. Numerical simulations of the complete mechanistic model with maximum-likelihood parameter estimates from best models for each group, from the start of bacterial exposure at *t* =−0.75 h. Vertical lines indicate *t* = 0 (end of the phagocytosis period), *t* = 1 h (first observation) and *t* = 9 h (second observation).

We then used our best-fitted model to predict the dynamics of the average number of bacteria per infected macrophage for each antibody subclass (figure 4). The comparison between the mean number of visible bacteria (figure 4*a*) and the mean number of replicating bacteria (figure 4*b*) reveals two striking predictions. Firstly, the relative abilities of the four specific antibody subclasses to control the number of replicating intracellular bacteria (with IgG3 being the most efficient and IgG2 being the least efficient) are in sharp contrast with their relative merits based on the number of visible bacteria (IgG3 resulting in the largest load and IgG2 the smallest). Secondly, the predicted numbers of replicating bacteria reach a plateau in all groups within a few hours, whereas the predicted numbers of visible bacteria keep increasing markedly for at least 9 h (figure 4*b*). To understand this apparent paradox, let us consider two cases. Our best model for IgG3 predicts that around 90 per cent of bacteria are able to replicate immediately after phagocytosis, causing the rapid accumulation visible in figure 4*a* and in the data; however, the relatively high value of *δ* means that these intracellular bacteria switch to a non-replicating stage on average within 1.5 h: as a result, the number of replicating bacteria at any time remains low (figure 4*b*) and the proportion of the visible bacteria actually replicating drops rapidly (figure 4*c*). At the other end of the spectrum, opsonization with IgG2 appears to be associated with around 50 per cent of refractory cells, keeping the average numbers of intracellular bacteria low (figure 4*a,b*); however, the proportion of those bacteria that are replicating keeps increasing (figure 4*c*) because there is no process of inactivation (*δ* = 0) in permissive cells.

Extending simulations beyond observed time points, the numbers of visible bacteria are expected to start decreasing after 10–12 h in the presence of IgG3 or IgG1. These differences are best summarized by considering the predicted proportions of intracellular bacteria that are replicating (figure 4*c*): while they increase to around 80 per cent within 9 h in the control and IgG2, the fractions of replicating bacteria drop to very low levels in the other three groups.

## 4. Discussion

This work demonstrates how mathematical and statistical models can be tailored to an experimental system to help formulate detailed predictions about complex biological processes that cannot be observed directly. We sought to reconstruct the dynamics of *S. enterica* infection at the cellular level, using human monocyte cultures and IgG antibodies. We previously reported variations in phagocytosis efficiency among IgG subclasses, owing to their different affinities for Fc receptors expressed at the surface of phagocytic cells [18]. However, opsonization has been shown to also affect the growth of pathogens inside infected cells [5–7]. By extending our previous *in vitro* gentamicin assay to 9 h post-infection and modelling the intracellular dynamics of *S. enterica*, we were able to quantify the impact of opsonization on bacterial replication and death in unprecedented detail. In particular, we showed that these effects are heterogeneous, as subpopulations of bacteria appear to stop replicating, and differ substantially between IgG subclasses. Our predictions will help design further experimental exploration of these intracellular dynamics.

Our first finding concerns the heterogeneity of intracellular replication of *S. enterica*. This was motivated by the observed distributions of bacteria within infected cells: after 9 h, each experimental group showed a peak ranging from four to around 15 bacteria, combined with a large but variable proportion of cells containing a single bacterium. Such bimodal distributions contrasted with previous observations in a murine model of typhoidal *S*. Typhimurium infection [26], and led us to hypothesize the existence of a subpopulation of non-replicating bacteria within infected cells. This phenomenon was reported recently in *S*. Typhimurium by Helaine *et al.* [23] in experimental infections of murine cells, based on measures of fluorescence dilution *in vitro.* However, the underlying mechanisms are still unknown, and we considered three simple scenarios. One assumption was that, following opsonization, an unknown proportion *p* of bacteria in the inoculum was inactivated (scenario (ii)). Alternatively, the heterogeneity might reside in the host cell population, so we allowed for a proportion *q* of ‘refractory’ cells that would completely prevent bacterial replication (scenario (iii)). Although scenario (iii) on its own does not allow cells to contain a mixture of replicating and non-replicating cells, contrary to Helaine *et al.*'s [23] observations, heterogeneities in phagocytic cell populations have been shown to play important roles in a related experimental system [13]. Finally, we considered the possibility that there was no intrinsic heterogeneity among the bacteria or the cells: by assuming that inactivation occurs randomly within all infected cells at a certain rate *δ*, we generated a model where an increasing proportion of intracellular bacteria are non-replicating (scenario (iii)). Because of our focus on intracellular bacterial replication, we chose to carry out experiments using a well-established gentamicin assay which kills extracellular bacteria. In the future, it would be interesting to use a different approach to bring together our predictions with those of Gog *et al.* [13] on phagocytosis dynamics.

Although each scenario on its own improved the goodness of fit (table 2) and resulted in bimodal distributions similar to those observed in the data, their relative support varied across opsonization groups. When allowing for combinations of scenarios, scenario (iii) came out on top for IgG2 and the control, whereas combining scenarios (i) and (ii) was best for IgG1, 3 and 4. Although the AIC analysis provides support for the alternative combination of scenarios (i) and (iii) with IgG4 and to a lesser extent with IgG1, there is no overall support for any single scenario across all opsonization groups. That IgG2 should cluster with the non-specific antibody used as a control is not unexpected: indeed IgG2 has been shown to have a much lower affinity to Fc receptors at the surface of phagocytic cells than the other subclasses [27]. While this is coherent with our estimates for phagocytosis rates (lowest for control and IgG2), our model suggests further consequences. The best-fitting model for the groups opsonized with IgG1, 3 or 4 assumes that opsonized bacteria are the source of heterogeneity; whereas the control group and the group opsonized with IgG2 were best described by the model with two subpopulations of host cells.

In addition to these qualitative differences relating to non-replicating bacteria, our model predicts variations among antibody subclasses in other parameters. Opsonization of various intra- or extracellular bacterial pathogens by immunoglobulins has been shown to enhance phagocytosis and killing by macrophages and neutrophils [28], but these processes have rarely been quantified properly: as demonstrated here, changes in bacterial numbers can be attributed to a combination of factors. According to our best models (figure 3), the anti-TSSPSAD antibodies caused limited increases in phagocytosis rate (*φ*) compared with the control group, with a maximum of 59 per cent for IgG1. In contrast, we predict that the same antibody increases the degradation rate of replicating bacteria (*d*) by 680 per cent. Interestingly, we found that all four subclasses enhanced in equal measure the replication rates of bacteria: even though there is no variation at all in the estimates of the baseline rate of bacterial replication (*a*), the model predicts a 50 per cent decrease in the rate at which bacterial replication slows down as they accumulate within cells (*b*). This means that, ignoring bacterial death and induction of the non-replicating state, we would expect a single infection event in a cell to produce on average seven bacteria within 9 h when opsonized with any of the four IgG subclasses, but only four bacteria when opsonized with the non-specific antibody. It is important to highlight that this predicted enhanced replication following opsonization by IgG is in part balanced by the concurrent inactivation of bacteria: in other words, we predict that a fraction of bacteria replicate faster following opsonization while a growing proportion stops replicating. As is visible from the data, the net result of opsonization in this particular experimental system is an accumulation of visible bacteria inside infected cells. Although we have no direct experimental evidence that the replication rate of *Salmonella* decreases as bacteria accumulate within a cell, both this study and an earlier one by Brown *et al.* [12], who analysed intracellular counts of bacteria from the livers of mice infected with *S*. Typhimurium, found statistical support for this phenomenon.

In order to test the validity of these predictions, further experiments would be required with the aim of detecting and quantifying non-replicating bacteria, possibly using fluorescence dilution methods. As shown in figure 4*c*, the model predicts clear, testable differences in the proportions of non-replicating bacteria among experimental groups, reflecting the two qualitatively different sets of scenarios selected by the statistical model. The parameter estimates also provide us with quantitative measures that might be validated experimentally. For example, the values of *p* (figure 3) represent predicted proportions of bacteria opsonized with each antibody subclass that are in a non-replicating state prior to phagocytosis—ranging from 0 to 40 per cent of all bacteria. In particular, it would be interesting to assess whether this correlates with variations in opsonization. We also predict that replicating bacteria are rapidly degraded inside cells (i.e. are no longer visible by microscopy, as described by parameter *δ* in our model), with median survival times ranging from 2 to 10 h depending on the antibody type used. Finally, the best model for the control and IgG2 groups assumes that the heterogeneity in bacterial replication is among the macrophage population: between 50 (IgG2) and 67 per cent (control) of cells are predicted to inhibit replication of phagocytized bacteria (parameter *q*). Heterogeneities in phagocytic cell populations are well documented; we recently quantified, using a similar modelling approach but a different experimental set-up, variability in susceptibility to infection within a population of murine bone-marrow derived macrophages exposed to *S*. Typhimurium [13]. However, the fact that we predict different proportions of refractory macrophages among experimental groups does not imply that we expect the cell samples used were different before the start of the experiment. Our interpretation (which remains to be tested experimentally) is that in these experimental conditions, a large proportion of cells can inhibit replication of bacteria with non-specific or IgG2 antibodies, whereas opsonization with IgG1 or IgG3 makes all cells initially permissive (*q* = 0), only to inhibit bacterial replication at a later point (with rate *δ*). Finally, our estimates of the degradation rate of non-replicating bacteria (*ɛ*) were lower than that of replicating bacteria (*d*), with half-lives ranging from 6 to 24 h among groups. Interestingly, even though differences in protocols preclude any formal quantitative comparisons, Helaine *et al.* [23] observed that non-replicating bacteria retained some biological functions for similar periods of time (namely 30–40% persistence after 24 h). We must underline, however, that the non-replicating bacteria, as defined in our model, could actually be dead: the only assumptions we make are that they cannot revert to a replicating state and that they are degraded (i.e. stop being visible by fluorescence microscopy) at a rate that can differ from that of replicating bacteria.

In conclusion, our multi-disciplinary approach provides new insight into the complex dynamics of bacterial infections. Using fluorescence microscopy with cultures, we were able to collect data from individual cells at two different time points, generating enough information to estimate multiple parameters and compare multiple scenarios. The statistical comparison of alternative scenarios that appeared plausible *a priori* provides objective ground to focus future experimental work on testing the specific hypotheses that are deemed more credible. Although this *in vitro* experimental model lacks many components of real-life infections—for example, T cell-mediated apoptosis facilitated by antibodies [29]—it has enabled us to draw inferences on a subset of essential processes, which in turn provides working hypotheses for *in vivo* experimental systems. Such multi-disciplinary studies, associated with more traditional approaches, can contribute to faster progress in our mechanistic understanding of infection dynamics.

## References

[1] Nowak MA 1997 Viral dynamics of primary viremia and antiretroviral therapy in simian immunodeficiency virus infection. J. Virol. 71, 7518–7525 9311831 10.1128/jvi.71.10.7518-7525.1997 PMC192098

[2] Grant AJ Restif O McKinley TJ Sheppard M Maskell DJ Mastroeni P 2008 Modelling within-host spatiotemporal dynamics of invasive bacterial disease. PLoS Biol. 6, e74 10.1371/journal.pbio.0060074 (doi:10.1371/journal.pbio.0060074) 18399718 PMC2288627

[3] Saenz RA 2010 Dynamics of infection and pathology in influenza. J. Virol. 84, 3974–3983 10.1128/JVI.02078-09 (doi:10.1128/JVI.02078-09) 20130053 PMC2849502

[4] Metcalf CJE 2011 Partitioning regulatory mechanisms of within-host malaria dynamics using the effective propagation number. Science 333, 984–988 10.1126/science.1204588 (doi:10.1126/science.1204588) 21852493 PMC3891600

[5] Edelson BT Unanue ER 2001 Intracellular antibody neutralizes listeria growth. Immunity 14, 503–512 10.1016/S1074-7613(01)00139-X (doi:10.1016/S1074-7613(01)00139-X) 11371353

[6] Joller N Weber SS Muller AJ Sporri R Selchow P Sander P Hilbi H Oxenius A 2010 Antibodies protect against intracellular bacteria by Fc receptor-mediated lysosomal targeting. Proc. Natl Acad. Sci. USA 107, 20 441–20 446 10.1073/pnas.1013827107 (doi:10.1073/pnas.1013827107) PMC2996673 21048081

[7] Uppington H Menager N Boross P Wood J Sheppard M Verbeek S Mastroeni P 2006 Effect of immune serum and role of individual Fcγ receptors on the intracellular distribution and survival of *Salmonella enterica* serovar Typhimurium in murine macrophages. Immunology 119, 147–158 10.1111/j.1365-2567.2006.02416.x (doi:10.1111/j.1365-2567.2006.02416.x) 16836651 PMC1782356

[8] Bronzan RN 2007 Bacteremia in Malawian children with severe malaria: prevalence, etiology, HIV coinfection, and outcome. J. Infect. Dis. 195, 895–904 10.1086/511437 (doi:10.1086/511437) 17299721

[9] Graham SM Molyneux EM Walsh AL Cheesbrough JS Molyneux ME Hart CA 2000 Nontyphoidal *Salmonella* infections of children in tropical Africa. Pediatr. Infect. Dis. J. 19, 1189–1196 10.1097/00006454-200012000-00016 (doi:10.1097/00006454-200012000-00016) 11144383

[10] Fields PI Swarson RV Haidaris CG Heffron F 1986 Mutants of *Salmonella typhimurium* that cannot survive within the macrophage are avirulent. Proc. Natl Acad. Sci. USA 83, 5189–5193 10.1073/pnas.83.14.5189 (doi:10.1073/pnas.83.14.5189) 3523484 PMC323916

[11] Richter-Dahlfors A Buchan AMJ Finlay BB 1997 Murine salmonellosis studied by confocal microscopy: *Salmonella typhimurium* resides intracellularly inside macrophages and exerts a cytotoxic effect on phagocytes *in vivo*. J. Exp. Med. 186, 569–580 10.1084/jem.186.4.569 (doi:10.1084/jem.186.4.569) 9254655 PMC2199036

[12] Brown SP Cornell SJ Sheppard M Grant AJ Maskell DJ Grenfell BT Mastroeni P 2006 Intracellular demography and the dynamics of *Salmonella enterica* infections. PLoS Biol. 4, e349 10.1371/journal.pbio.0040349 (doi:10.1371/journal.pbio.0040349) 17048989 PMC1609125

[13] Gog JR 2012 Dynamics of *Salmonella* infection of macrophages at the single cell level. J. R. Soc. Interface 9, 2696–2707 10.1098/rsif.2012.0163 (doi:10.1098/rsif.2012.0163) 22552918 PMC3427505

[14] Akeda H Mitsuyama M Tatsukawa K Nomoto K Takeya K 1981 The synergistic contribution of macrophages and antibody to protection against *Salmonella typhimurium* during the early phase of infection. J. Gen. Microbiol. 123, 209–214 7033456 10.1099/00221287-123-2-209

[15] Mastroeni P Villarreal-Ramos B Hormaeche CE 1993 Adoptive transfer of immunity to oral challenge with virulent salmonellae in innately susceptible BALB/c mice requires both immune serum and T cells. Infect. Immun. 61, 3981–3984 8359920 10.1128/iai.61.9.3981-3984.1993 PMC281103

[16] Lindow JC Fimlaid KA Bunn JY Kirkpatrick BD 2011 Antibodies in action: role of human opsonins in killing *Salmonella enterica* serovar Typhi. Infect. Immun. 79, 3188–3194 10.1128/IAI.05081-11 (doi:10.1128/IAI.05081-11) 21628517 PMC3147595

[17] Gondwe EN Molyneux ME Goodall M Graham SM Mastroeni P Drayson MT MacLennan CA 2010 Importance of antibody and complement for oxidative burst and killing of invasive nontyphoidal *Salmonella* by blood cells in Africans. Proc. Natl Acad. Sci. USA 107, 3070–3075 10.1073/pnas.0910497107 (doi:10.1073/pnas.0910497107) 20133627 PMC2840319

[18] Goh YS Grant AJ Restif O McKinley TJ Armour KL Clark MR Mastroeni P 2011 Human IgG isotypes and activating Fcγ receptors in the interaction of *Salmonella enterica* serovar Typhimurium with phagocytic cells. Immunology 133, 74–83 10.1111/j.1365-2567.2011.03411.x (doi:10.1111/j.1365-2567.2011.03411.x) 21323662 PMC3088969

[19] Riechmann L Clark M Waldmann H Winter G 1988 Reshaping human antibodies for therapy. Nature 332, 323–327 10.1038/332323a0 (doi:10.1038/332323a0) 3127726

[20] Redpath SM Michaelsen TE Sandlie I Clark MR 1998 The influence of the hinge region length in binding of human IgG to human Fcγ receptors. Hum. Immunol. 59, 720–727 10.1016/S0198-8859(98)00075-5 (doi:10.1016/S0198-8859(98)00075-5) 9796740

[21] Armour KL Clark MR Hadley P Williamson LM 1999 Recombinant human IgG molecules lacking Fcγ receptor I binding and monocyte triggering activities. Eur. J. Immunol. 29, 2613–2624 10.1002/(SICI)1521-4141(199908)29:08<2613::AID-IMMU2613>3.0.CO;2-J (doi:10.1002/(SICI)1521-4141(199908)29:08<2613::AID-IMMU2613>3.0.CO;2-J) 10458776

[22] Shen L Graziano RF Fanger MW 1989 The functional properties of FcγRI, II and III on myeloid cells: a comparative study of killing of erythrocytes and tumour cells mediated through the different Fc receptors. Mol. Immunol. 26, 959–969 10.1016/0161-5890(89)90114-4 (doi:10.1016/0161-5890(89)90114-4) 2531842

[23] Helaine S Thompson JA Watson KG Liu M Boyle C Holden DW 2010 Dynamics of intracellular bacterial replication at the single cell level. Proc. Natl Acad. Sci. USA 107, 3746–3751 10.1073/pnas.1000041107 (doi:10.1073/pnas.1000041107) 20133586 PMC2840444

[24] R Development Core Team 2012 R: a language and environment for statistical computing. Vienna, Austria: R Foundation for Statistical Computing

[25] Burnham KP Anderson DR 2001 Model selection and multimodel inference: a practical information-theoretic approach, 2nd edn. New York, NY: Springer

[26] Sheppard M Webb C Heath F Mallows V Emilianus R Maskell D Mastroeni P 2003 Dynamics of bacterial growth and distribution within the liver during *Salmonella* infection. Cell Microbiol. 5, 593–600 10.1046/j.1462-5822.2003.00296.x (doi:10.1046/j.1462-5822.2003.00296.x) 12925129

[27] Aase A Michaelsen TE 1994 Opsonophagocytic activity induced by chimeric antibodies of the four human IgG subclasses with or without help from complement. Scand. J. Immunol. 39, 581–587 10.1111/j.1365-3083.1994.tb03416.x (doi:10.1111/j.1365-3083.1994.tb03416.x) 8009174

[28] Silva MT 2010 Neutrophils and macrophages work in concert as inducers and effectors of adaptive immunity against extracellular and intracellular microbial pathogens. J. Leukoc. Biol. 87, 805–813 10.1189/jlb.1109767 (doi:10.1189/jlb.1109767) 20110444

[29] Eguchi M Kikuchi Y 2010 Binding of *Salmonella*-specific antibody facilitates specific T cell responses via augmentation of bacterial uptake and induction of apoptosis in macrophages. J. Infect. Dis. 201, 62–70 10.1086/648615 (doi:10.1086/648615) 19929376

